# Identification of novel variants in the *ARID1B* gene causing Coffin–Siris syndrome

**DOI:** 10.1007/s00431-025-06729-x

**Published:** 2026-01-17

**Authors:** Yan Ge, Xin-Yi Zhang, Xu Han, Jing-Tao Zhang, Wei-Meng Ma, Hao-Chun Yang, Hui-Qian Cao, Wei-Yu Lan, Wei Dong, Yang Hu, Yan-Ling Yang, Zhong-Sheng Sun, Ming Shen

**Affiliations:** 1 Institute of Genomic Medicine, Wenzhou Medical University, Wenzhou, 325000 China; 2 Hangzhou Institute of Medicine, Chinese Academy of Sciences, Hangzhou, 310000 Zhejiang Province China; 3 Children’s Medical Center, Peking University First Hospital, Beijing, 102600 China; 4 Fourth Clinical College of Henan Medical University, Xinxiang, 453000 Henan China; 5 Medical Innovation Research Division, Chinese PLA General Hospital, Beijing, 100853 China

**Keywords:** Coffin–Siris syndrome, *ARID1B*, Whole exome sequencing, Pedigree analysis

## Abstract

Coffin-Siris Syndrome (CSS) is a neurodevelopmental disorder caused by variants in genes encoding BRG1- and BRM-associated factor (BAF) chromatin-remodeling complex. *ARID1B* gene variants are the most common cause of CSS. This study aimed to identify novel pathogenic *ARID1B* variants in patients clinically diagnosed with CSS and to explore their pathogenic role. In this study, eight patients clinically diagnosed with CSS were enrolled, and whole exome sequencing (WES) was performed to identify potential pathogenic variants. Heterozygous variants in the *ARID1B* gene were identified in six patients, including one previously reported pathogenic nonsense variant and five novel pathogenic truncating variants. The combined annotation-dependent depletion (CADD) scores of the five novel variants were significantly above the mutation significance cutoff (MSC), suggesting their potential pathogenicity. According to the guidelines of the American College of Medical Genetics and Genomics (ACMG), these five novel variants were classified as pathogenic.

*Conclusions*: Our findings add five novel variants to the list of known pathogenic variants of the *ARID1B* gene. This study further clarifies an enhanced connection between *ARID1B* gene variants and CSS and expands the variant spectrum of CSS.

**Table:** 

What is Known: • Coffin–Siris syndrome (CSS) is a rare neurodevelopmental disorder characterized by developmental delay, intellectual disability, and hypoplasia of the fifth digits or nails. • Pathogenic variants in genes encoding subunits of the BAF chromatin-remodeling complex are the major genetic causes of CSS, with *ARID1B* being the most frequently mutated gene, and most variants of which are truncating and lead to haploinsuffucuency.
What is New: • Five novel heterozygous truncating variants in *ARID1B* were identified in eight patients clinically diagnosed with Coffin–Siris syndrome. • All novel variants showed high CADD scores and were classified as pathogenic according to ACMG guidelines.

What is Known:

• Coffin–Siris syndrome (CSS) is a rare neurodevelopmental disorder characterized by developmental delay, intellectual disability, and hypoplasia of the fifth digits or nails.

• Pathogenic variants in genes encoding subunits of the BAF chromatin-remodeling complex are the major genetic causes of CSS, with *ARID1B* being the most frequently mutated gene, and most variants of which are truncating and lead to haploinsuffucuency.

What is New:

• Five novel heterozygous truncating variants in *ARID1B* were identified in eight patients clinically diagnosed with Coffin–Siris syndrome.

• All novel variants showed high CADD scores and were classified as pathogenic according to ACMG guidelines.

## Introduction

Coffin-Siris syndrome (CCS, MIM: 135900) is a rare multi-system disability syndrome characterized by classic dysmorphic features, central hypotonia, developmental delay, intellectual disability and the underdevelopment of the fifth digit finger/toe or nail [1, 2]. Although it is inherited in an autosomal dominant manner, the pathogenic variants were de novo in most individuals. In 1970, Coffin and Siris first described the syndrome [3]. In 2012, it was found that variants of several genes encoding subunits of BAF complex could result in this syndrome [4, 5]. To date, compelling evidence demonstrates that CSS is caused by heterozygous pathogenic variants in *ARID1A*, *ARID1B*, *SMARCA2*, *SMARCA4*, *SMARCB1*, *SMARCE1*, *ARID2*, *DPF2*, *BICRA*, *PHF6*, *SMARCC2*, *SMARCD1*, *SOX11* and *SOX4* [5–12].

The estimated prevalence of CSS is approximately 1 in 100,000 individuals. The diagnosis of CSS is initially established based on characteristic clinical features and is subsequently confirmed by molecular genetic testing, which identifies a heterozygous pathogenic variant in one of the pathogenic genes [2]. When the individual presents with atypical phenotypic features, comprehensive molecular genetic testing, such as exome or genome sequencing, may be further required to establish a diagnosis. In the absence of longitudinal studies, the life span of CSS individuals is not available. Cases of children with CSS who succumb to aspiration pneumonia and/or seizures have been reported. However, one reported individual is alive at age 69 years, demonstrating that survival into late adulthood is possible [13–15]. CSS is not curable, and current treatment strategies focus on supportive care and symptom management [2].

The BAF complex is also referred to as the SWI/SNF complex and is a highly conserved ATP-dependent chromatin remodelling complex [16]. The BAF complex regulates gene expression by dynamically controlling nucleosome positioning and structure [9, 17]. Variants of several genes encoding subunits of BAF complex may be associated with cancer and neurodevelopmental disorders [18]. CSS is one of the syndromes commonly caused by mutations in BAF subunits [18, 19].

*ARID1B* is the most frequently pathogenic gene in CSS, accounting for 50–83% of all cases [10, 20]. *ARID1B* encodes AT-rich-interactive-domain-containing protein 1B (ARID1B). It is critical for neurite outgrowth and maintenance. The vast majority of pathogenic variants reported in *ARID1B* are truncating, predominantly leading to haploinsufficiency through nonsense-mediated mRNA decay [19, 21, 22].

In this study, WES was performed on eight patients clinically diagnosed with CSS to identify potential pathogenic variants. Heterozygous *ARID1B* variants were detected in six probands. Our findings expand the variant spectrum of CSS and provide additional evidence supporting the main role of *ARID1B* in CSS pathogenesis.

## Materials and methods

### Subjects

This project was approved by the Ethics Committee of the Chinese PLA General Hospital (Approval No. S2018-066–01). Informed consent was obtained from all patients and their parents prior to participation in the study. Genomic DNA was extracted from blood samples collected from patients and their parents according to standard protocols.

## WES and bioinformatic analysis

Genomic DNA was extracted from 200 µl of peripheral blood for all samples using the DNA Blood Midi/Minikit (Qiagen GmbH). The extracted DNA was subjected to quality control using agarose gel electrophoresis and UV spectrophotometry. Genomic DNA fragments were subjected to hybridization-based capture using the SureSelect Human All Exon V6 (Agilent DNA Technologies, Inc.) in accordance with the manufacturer’s protocol. Enrichment efficiency of the sequencing libraries was assessed via quantitative PCR, while library quality, including fragment size distribution and concentration, was evaluated using the Agilent Bioanalyzer 2100 system (Agilent Technologies, Inc.). Subsequently, high-throughput paired-end sequencing (150 bp) was performed on the Illumina NovaSeq 6000 platform using the NovaSeq Reagent Kit, with each sample sequenced at an input concentration of 300 pM.

After sequencing, quality control of raw data were performed using FastQC (v0.12.1), and aggregated reports were generated with MultiQC (v1.25.1). Adapter trimming and quality filtering were performed using Trim Galore (v0.6.10) with parameters set to a Phred quality score threshold of 25, minimum read length of 36 bp, maximum error rate of 0.1, and stringency of 3. Cleaned reads were aligned to the human reference genome GRCh38.p14 using BWA (v0.7.18) [23]. Subsequent processing was performed using GATK (v4.5.0.0) [24], including marking duplicates, fixing mate information, and base quality score recalibration using known variant databases. Variant calling was conducted with GATK HaplotypeCaller in GVCF mode for single samples, followed by joint genotyping using CombineGVCFs and GenotypeGVCFs. Variant quality score recalibration (VQSR) was applied separately to SNPs and indels using high-confidence training sets. Variant annotation was performed using ANNOVAR (v2020-0607) [25], integrating gene annotation, population frequency, pathogenicity databases, and functional prediction tools.

Primers for Sanger sequencing were designed using Primer5 software [26], and PCR amplification was performed following standard protocols. The PCR-amplified products were purified and further sent to Sangon Biotech Co., Ltd. (Shanghai, China) for bidirectional Sanger sequencing. Sequencing results were analyzed using DNAMAN software [27], and compared with the reference genome to confirm the presence and accuracy of the candidate variants.

## Results

### Clinical manifestations

Among the eight patients enrolled in this study, six patients carrying *ARID1B* variants are presented here, including three females (P1, P2, P6) and three males (P3, P4, P5). The age of these patients ranged from 0.8 to 6.6 years. Table 1 summarizes the detailed clinical features. All patients presented variable neurodevelopmental abnormalities. Developmental delay was clearly observed in three of the six patients (P1, P2, P5). Most patients (P3, P4, P5, P6) exhibited dyskinesia and speech delay, which is consistent with the typical neurobehavioral manifestations of CSS. Additional neurological and systemic manifestations included epilepsy (P1), hypotonia (P5, P6), and attention-deficit symptoms (P4, P6). Additionally, some patients presented with growth hormone deficiency (P1, P5), failure to thrive (P5), constipation (P1, P4, P6), and eczema (P3, P5), reflecting the multisystem characteristic of CSS. One female patient (P2) exhibited absence of carpal bone development. Another patient (P3) showed signs of renal tubular disorder accompanied by kidney stones and hypercalciuria, indicating metabolic abnormalities. Some cases also presented with characteristic CSS facial dysmorphism and mild genu varum (P5, P6).

**Table 1:** Clinical characteristics of six patients with *ARID1B* variants

Patient No	1	2	3	4	5	6
**Age (Y)**	4.1	0.8	2.5	5	3.8	6.6
**Gender**	F	F	M	M	M	F
**Neurological**						
Developmental delay	+	+	-	-	+	-
Epilepsy	+	-	-	-	-	-
Tics	+	-	-	-	-	-
Dyskinesia	-	-	+	+	+	+
Hypotonia	-	-	-	-	+	+
Speech delay	-	-	+	+	+	+
Attention-deficit	-	-	-	+	-	+
Sleep disturbances	-	-	+	-	-	-
**Kidney Anomalies**						
Kidney stones	-	-	+	-	-	-
Renal tubular disorder	-	-	+	-	-	-
Aminoaciduria	-	-	-	-	+	-
**Endocrine/Metabolic**						
Growth hormone deficiency	+	-	-	-	+	-
Secondary carnitine deficiency	+	-	-	-	-	-
Hypercarotenemia	+	-	-	-	-	-
failure to thrive	-	-	-	-	+	-
**Digestive/Liver**						
constipation	+	-	-	+	-	+
Liver dysfunction	-	-	+	-	-	-
Liver palpable below costal margin	-	-	-	-	-	+
**Skin**						
Eczema	-	-	+	-	+	-
**Skeletal**						
Absence of carpal bone development	-	+	-	-	-	-
Osteoporosis	-	-	-	-	+	-
Mild genu varum	-	-	-	-	-	+
**Other**						
Dysmorphic facial features	-	-	-	-	+	-

*M *male, *F *female, *Y *years

Auxiliary diagnostic evaluations revealed a series of metabolic and biochemical abnormalities (Table 2). These included abnormal levels of IGF-1 and growth hormone in P1, as well as abnormalities in phosphate, carnitine, and multiple amino acids in P5. Patient P5 also exhibited elevated levels of urinary organic acids, such as glutaric acid and 3-methylglutaric acid. In P6, urine toluidine blue was tested as positive, and blood ammonia levels were slightly elevated. No significant biochemical abnormalities were observed in patient P4. The clinical presentations of these cases highlight the complex multisystem disorder in CSS and emphasize the need for comprehensive clinical and biochemical assessments to guide diagnosis and management.

**Table 2:** Laboratory and auxiliary examination findings

	Patient 1	Patient 2	Patient 3	Patient 4	Patient 5	Patient 6
IGF-1	⬆	NA	NA	NA	NA	NA
Blood glucose	⬇	NA	NA	NA	NA	NA
GH	⬇	NA	NA	NA	NA	NA
Free carnitine	⬇	NA	NA	NA	NA	NA
Urinary ketone bodies	⬆	NA	NA	NA	NA	NA
Urinary dicarboxylic acids	⬆	NA	NA	NA	NA	NA
AST	NA	⬆	NA	NA	NA	NA
Uric acid/creatinine ratio	NA	NA	⬆	NA	NA	NA
Oxalate/creatinine ratio	NA	NA	⬆	NA	NA	NA
Glutaric acid	NA	NA	NA	NA	⬆	NA
Vitamin C	NA	NA	NA	NA	⬆	NA
3-methylglutaric acid	NA	NA	NA	NA	⬆	NA
Phosphate	NA	NA	NA	NA	⬆	NA
Multiple amino acids	NA	NA	NA	NA	⬆	NA
Free carnitine	NA	NA	NA	NA	⬇	NA
Total carnitine	NA	NA	NA	NA	⬇	NA
Urine toluidine blue	NA	NA	NA	NA	NA	+
Blood ammonia	NA	NA	NA	NA	NA	⬆

*IGF-1 *Insulin-like Growth Factor 1, *GH *Growth Hormone, *AST *Aspartate Aminotransferase

### Genetic findings

**Fig. 1:**
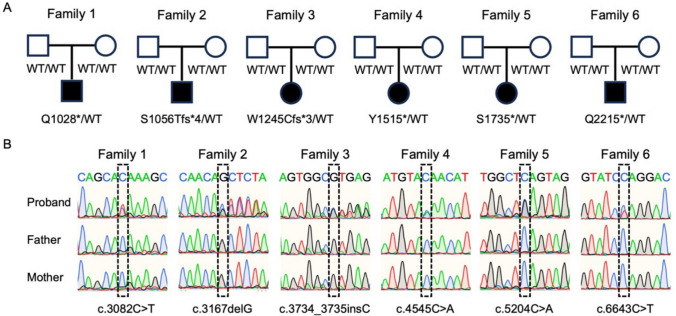
Pedigree structure and Sanger sequencing of six families (**A**) Pedigree diagrams of all six families in this study. (**B**) Sanger sequencing traces for six probands and their parents

WES was performed on all eight probands to identify potential pathogenic variants in 14 known CSS-associated genes. Truncating *ARID1B* variants were identified in six of the eight probands, while the remaining two probands carried known variants in other CSS-associated genes. In all six probands with *ARID1B* variants, these truncating variants were confirmed to be de novo by WES trio analysis (Fig. 1 A). Proband 1 carries a heterozygous of the *ARID1B* (NM_001374828.1): c.3082C > T (p.Q1028*) variant which leads to a change from glutamine to a premature stop codon at amino acid position 1028. This variant has been reported in ClinVar and is classified as pathogenic. Proband 2 and Proband 3 carry heterozygous frameshift variants: c.3167delG (p.S1056Tfs*4) and c.3734_3735insC (p.W1245Cfs*3), respectively. Both of these frameshift variants are highly likely to result in nonsense-mediated decay. Proband 4, 5, and 6 carry heterozygous nonsense variants in the *ARID1B* gene: c.4545C > A (p.Y1515*), c.5204C > A (p.S1735*), and c.6643C > T (p.Q2215*), respectively. All six variants were confirmed by Sanger sequencing (Fig. 1 B). The detailed characteristics of these variants are summarized in Table 3.

**Table 3:** Genetic characteristics of six CSS probands

Proband No	Gene (transcript)	Genomic variation	Protein variation	CADD Score	ClinVar database	ACMG pathogenicity level (Evidences)
1	*ARID1B* (NM_001374828.1)	c.3082C > T	p.Q1028*	39	pathogenic	pathogenic
2	*ARID1B* (NM_001374828.1)	c.3167delG	p.S1056Tfs*4	32	Not reported	Pathogenic (PVS1 + PS2 + PM2)
3	*ARID1B* (NM_001374828.1)	c.3734_3735insC	p.W1245Cfs*3	36	Not reported	Pathogenic (PVS1 + PS2 + PM2)
4	*ARID1B* (NM_001374828.1)	c.4545C > A	p.Y1515*	36	Not reported	Pathogenic (PVS1 + PS2 + PM2)
5	*ARID1B* (NM_001374828.1)	c.5204C > A	p.S1735*	41	Not reported	Pathogenic (PVS1 + PS2 + PM2)
6	*ARID1B* (NM_001374828.1)	c.6643C > T	p.Q2215*	40	Not reported	Pathogenic (PVS1 + PS2 + PM2)

*CADD *combined annotation-dependent depletion, *ACMG *The American College of Medical Genetics and Genomics

**Fig. 2:**
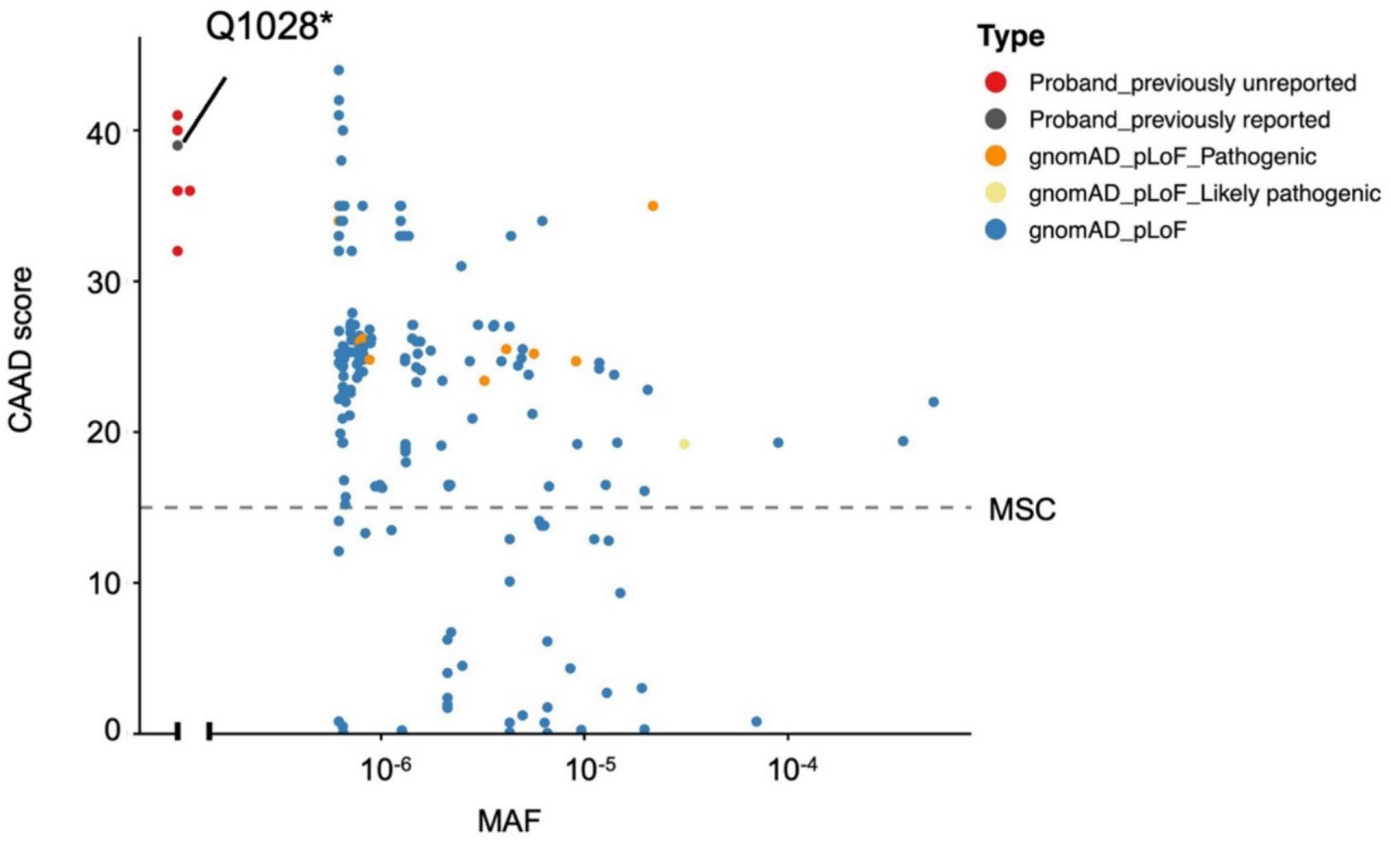
CADD MAF plot of all pLOF variants in *ARID1B* gene from the gnomAD database (blue). Orange dots represent pathogenic variants, yellow dots represent likely pathogenic variants, red dots represent previously unreported pLOF variants, and the gray dot represents previously reported variants identified in our CSS probands. MSC, mutation significance cutoff

These variants in probands 2–6 have not been reported in the public database like ClinVar (https://www.ncbi.nlm.nih.gov/clinvar/), the 1000 genomes project (1000G, https://www.internationalgenome.org/), the exome aggregation consortium (ExAC, https://gnomad.broadinstitute.org/) and the genome aggregation database (gnomAD v4.1.0, https://gnomad.broadinstitute.org/). All five predicted loss-of-function (pLOF, meaning stop-gain, frameshift, essential splice-site, and large insertion/deletion) variants are previously unreported. The CADD scores of all five variants were further calculated. By comparing them with the CADD scores of all pLOF sites in the *ARID1B* gene from the gnomAD database, we found that the CADD scores of these five variants were well above the MSC [28, 29], further suggesting that they are deleterious (Fig. 2).

According to the ACMG [30], the following variants in the *ARID1B* gene: c.3167delG (p.S1056Tfs*4), c.3734_3735insC (p.W1245Cfs*3), c.4545C > A (p.Y1515*), c.5204C > A (p.S1735*), and c.6643C > T (p.Q2215*) were classified as pathogenic. According to ClinGen (https://search.clinicalgenome.org/CCID:004169), truncating variants in *ARID1B* cause haploinsufficiency and are definitively linked to CSS. Therefore, the novel pLoF variants fulfilled the PVS1 criterion. The WES analysis of trios confirmed that these variants were de novo variants, and fulfilled the PS2 criterion. In addition, these variants were absent from population database such as gnomAD, meeting the PM2 criterion. The combination of these criteria provides support for classification of these variants as pathogenic.

## Discussion

Variants in the *ARID1B* gene are the most common cause of CSS. In this study, among the eight patients clinically diagnosed with CSS, six patients carrying *ARID1B* variants were analyzed in detail. A total of six truncating variants were detected, including four nonsense variants and two frameshift variants, five of which were novel. In accordance with previous studies, all of these variants were truncating. According to the ACMG guidelines, we classified the five novel de novo variants as pathogenic.

*ARID1B* haploinsufficiency is considered as the primary pathogenic mechanism of the CSS. In previous studies, only a few *ARID1B* variants were classified as pathogenic based on clinical data and their de novo occurrence. Most of the remaining variants were considered to be of uncertain significance. Studies have shown that *ARID1B* haploinsufficiency disrupts normal regulation of the cell cycle, thereby interfering with cell differentiation and development, leading to the onset of related diseases [31].

*ARID1B* is not only the most frequently mutated gene in CSS, but also one of the common genes mutated in intellectual disability (ID), with an extremely broad phenotypic spectrum. Due to the fact that the BAF complex is composed of more than 25 core and interchangeable protein subunits, which can give rise to functionally distinct and cell type–specific complexes, variations in these components are likely to contribute to the phenotypic diversity of *ARID1B*-related disorders [32]. A study showed that *ARID1B*-CSS patients frequently exhibit typical CSS features, Facial and limb features include thick eyebrows, long eyelashes, broad nasal wings, a long and/or wide philtrum, small nails, hypoplasia or absence of the fifth distal phalanx, and hypertrichosis. In contrast, *ARID1B*-ID patients presented a series of features such as myopia, cryptorchidism, sleep apnea, attention-deficit hyperactivity disorder, and a high pain threshold [33]. In our cohort of six probands, some typical CSS features were observed. Facial and limb features included dysmorphic facial characteristics (P5), whereas most patients exhibited neurodevelopmental features, including developmental delay (P1, P2, P5), dyskinesia (P3–P6), speech delay (P3–P6), and hypotonia (P5, P6). These findings highlight the variable expressivity of *ARID1B*-associated CSS, with partial manifestation of classic features in each patient. Therefore, the definitive diagnosis of CSS requires an integrated approach combining clinical phenotypic evaluation with molecular genetic testing, particularly the identification of pathogenic variants in *ARID1B* and other related genes.

In conclusion, six CSS probands carrying *ARID1B* variants were analyzed, and comprehensive clinical evaluations were performed using multiple parameters. WES was conducted on the probands and their parents to identify pathogenic variants. Our findings expand the mutational spectrum of *ARID1B*-associated CSS and provide additional evidence to support genetic counseling for affected families.

## Data Availability

The raw sequence data reported in this paper have been deposited in the Genome Sequence Archive (Genomics, Proteomics & Bioinformatics 2021) in National Genomics Data Center (Nucleic Acids Res 2025), China National Center for Bioinformation/Beijing Institute of Genomics, Chinese Academy of Sciences (GSA-Human: HRA003652) that are publicly accessible at [https://ngdc.cncb.ac.cn/gsa-human](https:/ngdc.cncb.ac.cn/gsa-human) [34, 35].
